# The Effect of Artemisinin on Inflammation-Associated Lymphangiogenesis in Experimental Acute Colitis

**DOI:** 10.3390/ijms21218068

**Published:** 2020-10-29

**Authors:** Ae Sin Lee, Haeng Jeon Hur, Mi Jeong Sung

**Affiliations:** Research Group of Natural Materials and Metabolism, Korea Food Research Institute, Jeollabuk-Do 55365, Korea; aslee@kfri.re.kr (A.S.L.); mistltoe@kfri.re.kr (H.J.H.)

**Keywords:** artemisinin, lymphangiogenesis, inflammatory bowel disease, experimental acute colitis

## Abstract

Inflammatory bowel disease (IBD) is characterized by inflammation, angiogenesis, and lymphangiogenesis. Artemisinin (Art), a chemical compound isolated from *Artemisia annua* L. (sweet wormwood), has several biochemical properties including antibacterial, anticancer, anti-inflammation, and anti-angiogenesis effects. We investigated the effects of Art on inflammation-induced lymphangiogenesis in a dextran sulfate sodium (DSS)-induced mouse acute colitis model. The mice were orally administered Art for 7 days before being evaluated using the disease activity index (DAI) and documenting colonic inflammatory changes, colon edema, microvessel density, lymphatic vessel density (LVD), proinflammatory cytokine levels, and vascular endothelial growth factor (VEGF)-C and VEGF-D/VEGF receptor (VEGFR)-3 mRNA expression levels in colon tissue. Art reduced DSS-induced lymphatic vessel endothelial hyaluronan receptor-1-positive LVD. Art also reduced the symptoms of colitis, improved tissue histology, and relieved inflammatory edema in mice affected by colitis. In addition, Art decreased the infiltration of immunomodulatory cells and inflammatory cytokines, which involved reduction of VEGF-C, -D, and VEGFR-3 expression. Taken together, our findings suggest that Art ameliorates inflammation-driven lymphangiogenesis in an experimental colitis mouse model via the VEGF-C/VEGFR-3 signaling pathway, implicating this pathway as a potential target for the treatment of IBD.

## 1. Introduction

Inflammatory bowel disease (IBD), including Crohn’s disease and ulcerative colitis, is characterized by chronic inflammatory disorders, arising as a result of the interaction between environmental and genetic factors leading to immunological responses and inflammation [1,2]. Lymphangiogenesis, the formation of new lymphatic vessels from pre-existing lymphatic vessels, facilitates inflammation under conditions such as cancer metastasis, chronic inflammation, and transplant rejection [3,4]. The microvasculature drives the pathophysiology of IBD by contributing to mucosal inflammation through ineffective tissue perfusion, altered angiogenesis, and lymphangiogenesis, resulting in tissue reorganization [5]. Recently, meaningful increases in blood and lymphatic vessel density (LVD) have been demonstrated in an experimental colitis mouse model [6,7]. In addition, patients with IBD consistently show enlargement or dilation of the mucosa resulting from lymphatic obstruction and submucosal edema [8]. 

Vascular endothelial growth factor (VEGF)-C and VEGF-D are related to VEGF receptor (VEGFR)-3 through lymphatic vessels and lymphangiogenesis. Enhancing inflammatory lymphangiogenesis by VEGF-C aggravated intestinal inflammation in experimental colitis mice [9,10]. Recently, Sato et al. [2] reported that anti-lymphatic treatment with anti-VEGFR-3 antibodies decreased inflammation and submucosal edema, increased leukocyte infiltration, and caused the lymphatic vessels to become tortuous. These studies indicate that VEGF-C/VEGFR-3 are required for inflammation-associated lymphangiogenesis and the immune response [11]. Therefore, improving lymphatic vessels through VEGF-C may provide a potential strategy for the treatment of the inflammatory condition in IBD.

Artemisinin (qinghaosu; Art), isolated from *Artemisia annua* L. (sweet wormwood), is well known in the prevention and treatment of malaria [12,13]. Art and its derivatives exhibit several biochemical properties including anticancer, anti-inflammation, and anti-oxidative stress effects [14,15]. In addition, Art has anti-angiogenic properties [16], and one of its derivatives inhibits corneal neovascularization [17]. Moreover, Hu et al. [18] reported that Art protects against dextran sulfate sodium (DSS)-induced IBD, which is associated with the activation of the pregnane X receptor, a steroid and xenobiotic-sensing nuclear receptor. However, until now, there has been no report on the anti-lymphatic effect of Art in IBD. Based on the findings presented above, it is reasonable to hypothesize that the anti-lymphatic activity of Art may affect inflammation and disease pathology related to various chronic inflammatory conditions. To investigate this possibility, we examined the effects of Art on lymphangiogenesis and the immune response through VEGF-C/VEGFR-3 signaling using a DSS-induced experimental colitis mouse model. Our findings provide clues to the potential of Art as a new therapeutic tool in targeting inflammatory lymphangiogenesis.

## 2. Results

### 2.1. Art Reduces Inflammatory Lymphangiogenesis in Mice with DSS-Induced Colitis

**Figure 1:**
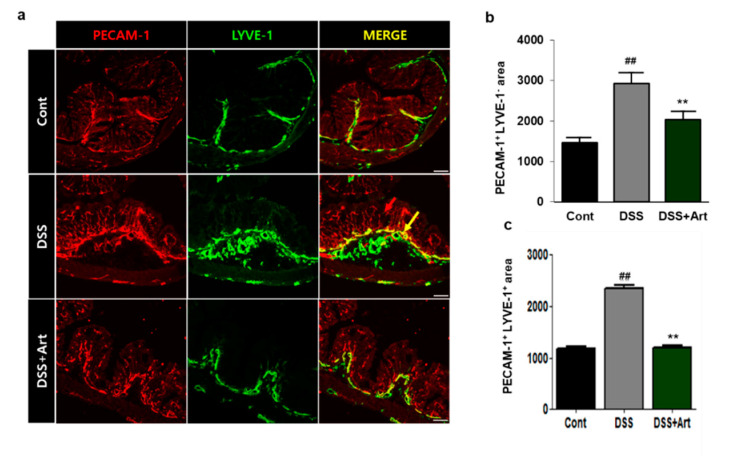
Immunofluorescent analysis of platelet endothelial cell adhesion molecule (PECAM-1) and lymphatic vessel endothelial hyaluronan receptor ( LYVE-1) staining in control mice (Cont), mice with dextran sulfate sodium (DSS)-induced colitis, and mice treated with DSS + artemisinin (Art). (**a**) The colon tissue was stained using PECAM-1 (red) and LYVE-1 (green), and PECAM-1/LYVE-1. Scale bar, 50 μm. PECAM^+^ LYVE-1^−^ (red arrow) indicates the blood vessels, and PECAM^+^ LYVE-1^+^ indicates the lymphatic vessels (**b**) Quantification of the area of PECAM^+^ LYVE-1^−^ blood vessels (**c**) Area with PECAM^+^ LYVE-1^+^ lymphatic vessels in colon tissue. Data shown are from three independent experiments and are expressed as mean ± SD (*n* = 10 per group); ^##^ *p* < 0.01, DSS versus Cont; ** *p* < 0.01, DSS + Art versus DSS.

To investigate the effects of Art on lymphangiogenesis, we stained colon tissue sections for platelet endothelial cell adhesion molecule (PECAM-1) and lymphatic vessel endothelial hyaluronan receptor (LYVE-1) (Figure 1a). Compared to control mice, the DSS-treated mice had significantly increased LYVE-1-positive/PECAM-1-positive lymphatic vessel densities in the submucosal layer (*p* < 0.01), whereas the addition of Art resulted in a significant decrease in vessel density relative to that observed after treatment with DSS alone (*p* < 0.01) and similar to that of the control (Figure 1b). These results suggest that Art suppresses DSS-induced lymphangiogenesis.

### 2.2. Art Attenuates the Symptoms of Colitis, Improves Tissue Histology, and Relieves Inflammatory Edema in Mice with Colitis

**Figure 2:**
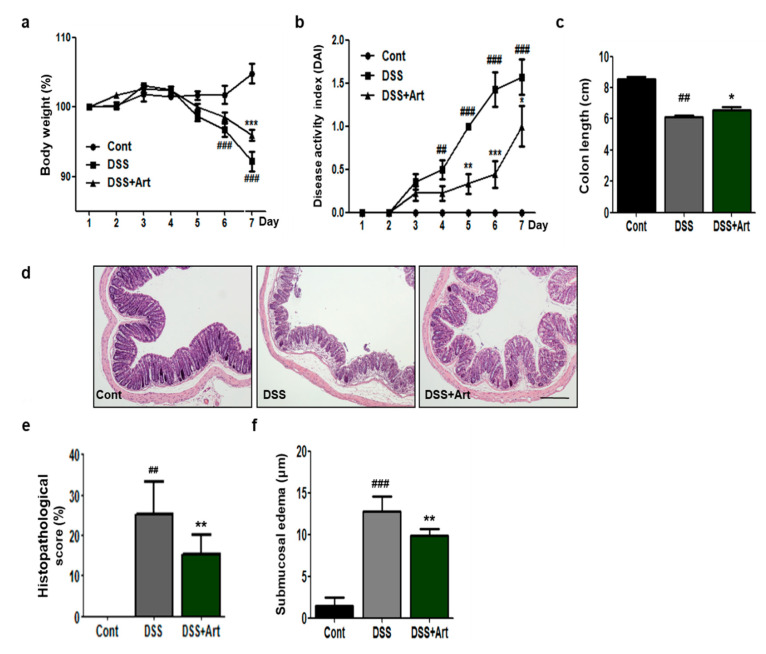
Clinical symptomatic parameters in mice with DSS-induced colitis and effect of Art. (**a**) Changes in body weight over time relative to day 1, (**b**) changes in clinical scores for control (Cont), DSS, and DSS + Art mice, (**c**) length of colon collected from mice 7 days after commencement of DSS administration, (**d**) histopathological examination of colon tissue obtained 7 days after the commencement of DSS administration, stained with hematoxylin and eosin. Cont (no DSS; control), DSS (3% DSS), DSS + Art (3% DSS + artemisinin at 20 mg/kg body weight per day). Original magnification: 40×, (**e**,**f**) histopathological score and submucosal edemas for the analyzed slides. Data shown are from three independent experiments and are expressed as mean ± SD (*n* = 10 per group); ^##^ *p* < 0.01 and ^###^ *p* < 0.001 DSS versus Cont; * *p* < 0.05, ** *p* < 0.01, and *** *p* < 0.001 DSS + Art versus DSS.

To examine the effects of Art on colonic inflammation, we measured clinical symptomatic parameters, such as body weight loss, disease activity index (DAI), and shortening of the colon in DSS-induced colitis mice. We observed a significant loss in body weight in DSS-treated mice compared to control mice on day 6; however, the DSS + Art mice lost significantly less body weight compared to DSS-treated mice on day 7 (*p* < 0.001; Figure 2a). The DAI score increased significantly for DSS-treated mice compared to control mice, but artemisinin treatment significantly reduced this score by day 5 (*p* < 0.01; Figure 2b). The shortening of the colon is an index of disease severity in colitis. We measured the increase in colon length in the DSS + Art group compared to DSS-treated mice (*p* < 0.05; Figure 2c). Further, analysis of hematoxylin and eosin (H&E) tissue staining revealed acute inflammation characterized by ulceration, crypt dilation, and goblet cell depletion, as well as cell infiltration by macrophages and lymphocytes in the DSS-treated group. However, Art administration to DSS-induced colitis mice noticeably reduced cell infiltration and repressed mucosal injury and edema (Figure 2d, Figure S1). Compared to control mice, DSS-treated mice had significantly higher histological scores of inflammation (*p* < 0.01) and inflammatory edema (*p* < 0.001), which were effectively reduced by Art treatment (*p* < 0.01; Figure 2e,f). Therefore, these data indicate that Art probably has an inhibiting effect on DSS-induced inflammation.

### 2.3. Art Decreases the Infiltration of Immunomodulatory Cells during Colitis

**Figure 3:**
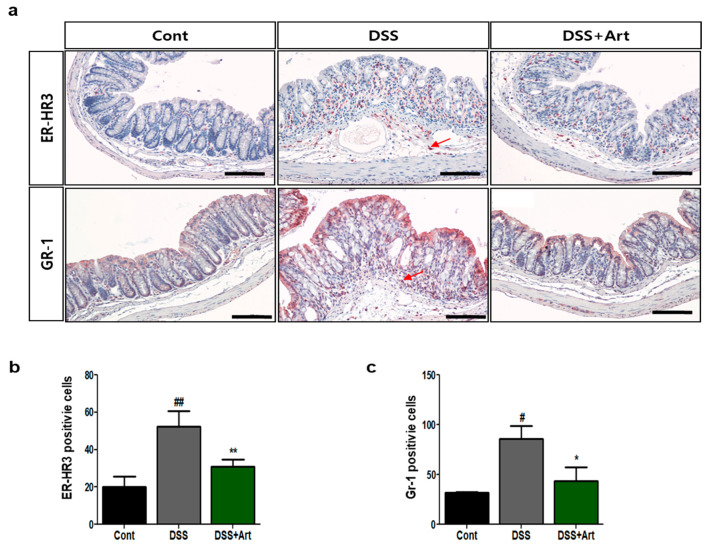
Immunohistochemical analysis of ER-HR3 and GR-1 staining of colon tissue. (**a**) Representative images of paraffin-embedded colon sections from control mice (Cont), mice with DSS-induced colitis (DSS), and mice treated with DSS + artemisinin (DSS + Art) after staining with anti-ER-HR3 or Gr-1 antibodies, used to count ER-HR3- and Gr-1-positive cells. The arrow indicates positive cells. Scale bar, 50 μm. (**b**) Quantification of ER-HR3-positive and (**c**) Gr-1-positive cells in colon tissue. Data shown are from three independent experiments (five randomly selected fields, ×200) and are expressed as mean ± SD (*n* = 10 per group); ^#^ *p* < 0.05 and ^##^ *p* < 0.01 DSS versus Cont; * *p* < 0.05, ** *p* < 0.01 DSS + Art versus DSS.2.4. Art reduces the expression of lymphangiogenic factors in the DSS-induced colitis model.

To investigate whether Art modulates the infiltration of leukocytes in mice with DSS-induced colitis, we counted the number of ER-HR3^+^ macrophages and Gr-1^+^ neutrophils in the three groups of mice. Concurrent treatment with Art resulted in a marked decrease in DSS-driven infiltration of ER-HR3^+^ macrophages and Gr-1^+^ neutrophils (*p* < 0.01 and *p* < 0.05, respectively; Figure 3). Therefore, Art may regulate DSS-induced infiltration of immunomodulatory cells, which is observed in inflammation.

**Figure 4:**
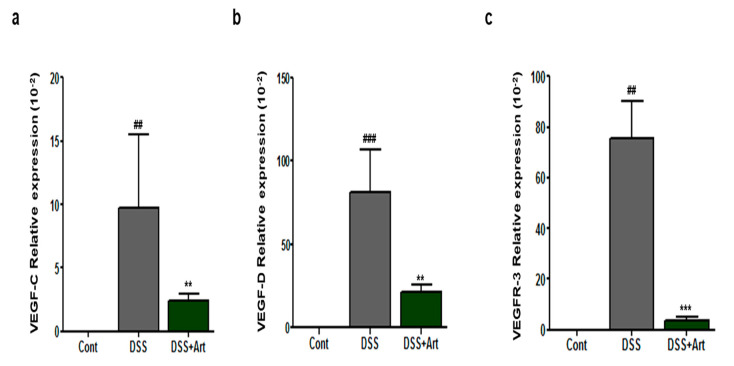
Quantitative real-time PCR analysis of vascular endothelial growth factor (VEGF)-C (**a**), VEGF-D (**b**), and VEGF receptor (VEGFR)-3 (**c**) expression in control mice (Cont), mice with DSS-induced colitis (DSS), and mice treated with DSS + artemisinin (DSS + Art). RNA was extracted from isolated colon tissue 7 days after the commencement of DSS administration. Data shown are from three independent experiments and are expressed as mean ± SD (*n* = 10 per group); ^##^ *p* < 0.01 and ^###^ *p* < 0.001 DSS versus Cont; ** *p* < 0.01 and *** *p* < 0.001 DSS + Art versus DSS.

VEGF-C and VEGF-D are lymphangiogenic factors that can induce lymphangiogenesis by binding to VEGFR-3 [19]. We explored the levels of VEGF-C, -D, and VEGFR-3 in colon sections by real-time polymerase chain reaction (PCR) analysis. As expected, DSS treatment increased VEGF-C, -D, and VEGFR-3 levels in colitis mice, whereas concurrent treatment with Art clearly decreased the DSS-induced increase in VEGF-C (*p* < 0.01), VEGF-D (*p* < 0.01), and VEGFR-3 (*p* < 0.001) mRNA levels (Figure 4a–c). Therefore, we concluded that Art regulates lymphangiogenesis mediated by VEGF-C, -D, and VEGFR-3 in DSS-induced colitis mice.

### 2.4. Art Reduces the Expression of Proinflammatory Cytokines in Mice with DSS-Induced Colitis 

**Figure 5:**
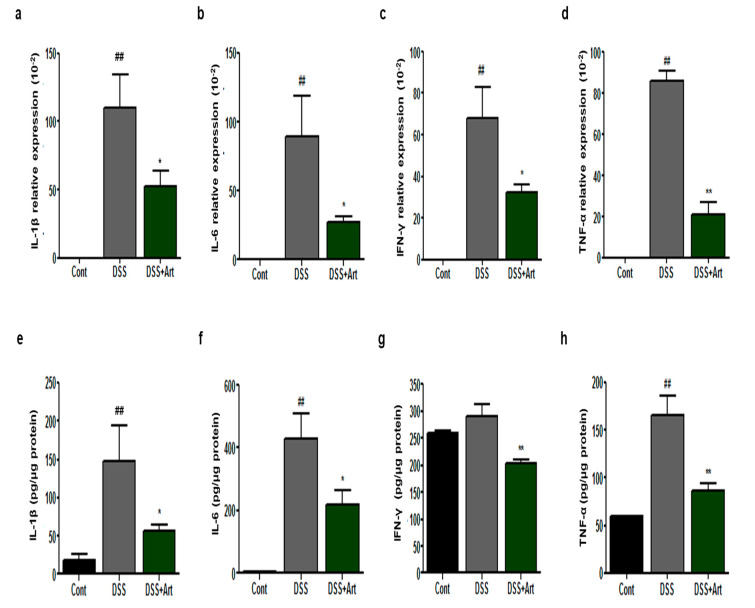
Colonic mRNA levels and expression of inflammatory mediators in control mice (Cont), mice with DSS-induced colitis (DSS), and mice treated with DSS + artemisinin (DSS + Art) 7 days after the commencement of DSS administration. (**a**–**d**) Results of RT-qPCR for RNA extracted from colon tissue. (**e**–**h**) Inflammatory cytokine levels in colonic tissue extracts. Data shown are from three independent experiments and are expressed as mean ± SD (*n* = 10 per group); ^##^ *p* < 0.01 DSS versus Cont; * *p* < 0.05 and ** *p* < 0.01 DSS + Art versus DSS.

Increased levels of proinflammatory cytokines activating immune cells is a feature of colitis [20]. We investigated whether Art treatment could inhibit the levels and expression of proinflammatory cytokines in colonic tissues. mRNA expression levels of the proinflammatory cytokines interleukin (IL)-1β, IL-6, interferon (IFN)-γ, and tumor necrosis factor (TNF)-α (*p* < 0.01) increased in DSS-treated mice compared with control mice (Figure 5a–d). However, mice treated with Art showed a markedly suppressed level of mRNA expression in all cases (*p* < 0.05 Figure 5a–c, and *p* < 0.001 Figure 5d). In addition, the protein levels of inflammatory cytokines in mice treated with Art were significantly lower than those in DSS-treated mice (*p* < 0.05 Figure 5e,f, and *p* < 0.001 Figure 5g,h). These data suggest that the anti-inflammatory effects of Art are likely associated with its ability to regulate the expression and release of cytokines.

### 2.5. Art Improves Lymph Flow in DSS-Induced Colitis

**Figure 6:**
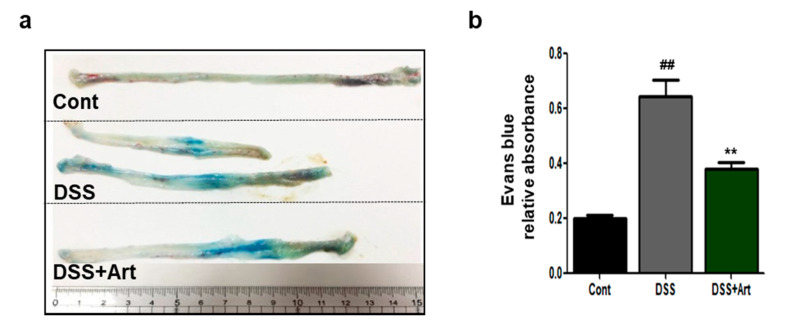
Evans blue dye assay was used to analyze lymphatic drainage from the colon of control mice (Cont), mice with DSS-induced colitis (DSS), and mice treated with DSS + artemisinin (DSS + Art) 7 days after the commencement of DSS administration. (**a**) Distribution of Evans blue dye in the distal colon of the three mice groups. (**b**) Quantification of distal colon lymphatic drainage of Evans blue dye. Data shown are from three independent experiments and are expressed as mean ± SD (*n* = 10 per group); ^##^ *p* < 0.01, DSS versus Cont; ** *p* < 0.01 DSS + Art versus DSS.

To investigate whether Art affects lymphatic drainage in DSS-induced colitis, we performed an Evans blue dye assay and observed that the dye was extravasated from dilated vessels in the colon (Figure 6). Furthermore, lymphatic drainage increased significantly in the DSS-treated group compared to the control group (*p* < 0.01), but treatment with Art reduced the amount of leaked Evans blue dye from the colon compared to DSS treatment alone (*p* < 0.01; Figure 6b). These results indicate that Art treatment reduces lymphatic drainage in DSS-induced colitis.

## 3. Discussion

Art is widely known as a first-line antimalarial drug. Recently, growing evidence has shown that Art is an effective treatment for various diseases, showing anti-cancer, anti-inflammatory, and anti-angiogenesis activities [16,17,18]. However, the preventive or therapeutic effect of Art on colitis remains unclear. The present study shows that Art attenuates inflammation-driven lymphangiogenesis in a mouse model of DSS-induced colitis. IBD patients and experimental colitis mouse models are representatives of lymphangiogenesis [21]. Recently, inflammation-associated lymphangiogenesis was shown to be related to the pathogenesis of intestinal inflammation. The regulation of the function of lymphatic vessels via the control of key signaling pathways could improve intestinal inflammation. Therefore, in this study, we investigated the effect of the natural compound Art on lymphangiogenesis-associated inflammation in experimental colitis. 

IBD includes several chronic inflammatory disorders of the intestine and colon, in which the patient suffers from severe diarrhea, abdominal pain, and weight loss due to rectal bleeding. It contributes to chronic inflammation through inflammatory cell infiltration and mucosal edema, leading to tissue remodeling [22,23]. In this study, we found that Art suppressed the DSS-induced symptoms of colitis, including weight loss, shortening of the colon, and increased DAI score. In addition, Art reduced tissue injury, such as infiltration of inflammatory cells and epithelial disruption and edema (Figure 2), suggesting that Art attenuates DSS-induced colitis.

Several lines of evidence have demonstrated that IBD results in an increase in blood vessel density, dilation of blood vessels and lymphatic vessels, and lymphatic expansion [9]. In an inflamed colon, the increase in blood vessel density and dilation leads to leakiness and is responsible for triggering the release of growth factors. These, in turn, recruit additional inflammatory cells, resulting in lymphatic drainage. An increase in lymphatic vessels compared to blood vessels causes inflammatory edema and infiltration of neutrophils, which leads to lymphatic dysfunction [8]. In this study, Art suppressed DSS-induced blood vessel density and LVD (Figure 1). Therefore, Art ameliorates DSS-induced colitis injury through the suppression of angiogenesis and lymphangiogenesis

Inflammation-associated lymphangiogenesis contributes to the production of proinflammatory cellular mediators, such as macrophages, neutrophils, and dendritic cells [24,25]. Activated macrophages, stimulated by proinflammatory cytokines, produce inflammatory-associated factors, such as VEGF-C and -D [25]. In our study, Art significantly suppressed the DSS-induced increase in macrophages, neutrophils (Figure 3), and proinflammatory cytokines, such as TNF-α, IL-1β, and IL-6. In addition, VEGF-C and -D promote new lymphatic vessels. VEGF-A indirectly amplifies lymphatic vessel production by selecting macrophages that secrete VEGF-C and -D, which are characterized as major pro-lymphangiogenic factors [26,27]. Activated VEGF-C and -D have increased affinity for VEGFR-3 and have the ability to bind directly to VEGFR-2 [19]. Recently, D’Alessio et al. [10] showed that VEGF-C/VEGFR-3 controls lymphatic function and inflammatory activity in experimental colitis models. In our previous study, we reported that COMP-angiopoietin-1 reduces the expression of VEGF-C and -D mRNA in colitis [7]. In the current study, Art inhibited the increased mRNA expression of VEGF-C and -D and VEGFR-3 in mice with DSS-induced colitis (Figure 4). Collectively, these results demonstrate that Art not only controls the regulation of inflammation-associated lymphangiogenesis through the VEGF-C-and -D signaling pathway during intestinal inflammation, but also suppresses the secretion of proinflammatory cytokines and, hence, activated macrophages. 

The pathogenesis of IBD includes the impaired clearance of foreign material, leading to impaired lymph drainage and sustained activation of innate immune cells [28]. Inhibition of lymphangiogenesis in IBD is controlled to decrease lymphatic drainage and reduce inflammatory cell mobilization. In our study, we found that Art reduced vascular permeability (Figure 6). These results demonstrate that Art could modulate lymphatic clearance function. Using a DSS-induced acute colitis mouse model, we demonstrated in this study that Art significantly attenuates intestinal inflammation by suppressing lymphatic drainage and reducing edema, inflammatory cell infiltration, and proinflammatory cytokines levels. In addition, Art prevented lymphatic vessel increase by reducing lymphangiogenic factors, such as VEGF-C and VEGF-D, and their receptor VEGFR-3. Therefore, Art has significant potential as a therapeutic agent in the prevention and targeted treatment of conditions modulated by mucosal inflammation-associated lymphangiogenesis.

## 4. Materials and Methods

### 4.1. Animal Model

Thirty-eight-week-old male C57BL/6 mice were purchased from Charles River Korea (Seoul, Korea). The mice were maintained with free access to water and food at a temperature of 21–25 °C and 40–60% humidity in a constant light/dark cycle. All experimental animals were treated and maintained according to the regulations on the management and use of experimental animals by the Korea Food Research Institute (KFRI). The experimental protocols were carried out in accordance with the Institutional Animal Care and Use Committee of the KFRI (KFRI-IACUC, KFRI-M-18002). To induce acute colitis, 3% DSS (molecular weight 36‒50 kDa; MP Biochemicals, Aurora, OH, USA) was dissolved and fed to mice in their drinking water. For the experiments, colitis mice in the control (Cont) group (*n* = 10) received only fresh drinking water without DSS, while mice in the DSS group (*n* = 10) were given DSS dissolved in drinking water and administered orally with a corn oil vehicle for 7 days. Mice in the DSS + Art group (*n* = 10) received oral administration of 20 mg/kg Art simultaneously with DSS-infused drinking water for 7 days. On day 7 of DSS administration, all mice were euthanized by CO_2_ inhalation, and blood samples and colon tissue were collected.

### 4.2. Assessment of Disease Severity

The DAI was evaluated as a combined score by observing daily weight loss, stool status, and the degree of bloody stool after the initiation of DSS-induced colitis. The DAI scores were evaluated using a previously described scoring system [29]. Scores were defined by change in weight (0: <1%, 1: 1–5%, 2: 5–10%, 3: 10–15%, and 4: >15%); blood (0: negative, 2: positive, and 4: gross bleeding); and stool form (0: normal, 2: loose stools, and 4: diarrhea).

### 4.3. Histopathological Analysis

Distal colon tissues were fixed in 4% formaldehyde and embedded in paraffin blocks. Paraffin-embedded colon tissue sections were stained on slides with H&E for histopathological analysis. The sections were evaluated for histological changes under an optical microscope (×40 magnification) with different fields randomly selected according to colitis severity, using the scoring methods detailed above [30]. After H&E staining, the width of the colon submucosa (inflammatory edema) was measured in five randomly selected non-overlapping fields under light microscopy by two different observers (×10 magnification). 

### 4.4. Immunofluorescence Analysis

Frozen colon specimens were sectioned (8 μm thick) for the observation of blood and lymphatic vessels. We used anti-mouse LYVE-1 monoclonal antibodies (Angiobio, Del Mar, CA, USA), anti-mouse PECAM monoclonal antibodies (Chemicon, Temecula, CA, USA), anti-mouse F4/80 monoclonal antibodies (eBioscience, San Diego, CA, USA), and Cy3- and FITC-conjugated antibodies for immunofluorescence analysis. Images of stained sections were taken with a Nikon Eclipse Ti confocal microscope (Tokyo, Japan). Vessel area in each colon specimen was analyzed as the PECAM-1^+^ and LYVE-1^+^ area of the total area using Image J software based in Fiji, at a magnification of ×200 in 5 regions, each amounting to a 0.21 m^2^ area, by two different observers.

### 4.5. Immunohistochemistry Analysis

Immunohistochemical staining was performed as previously described [31]. Slides containing colon tissue were incubated with anti-mouse ER-HR3 monoclonal antibody (BMA Biomedicals, Rheinstrasse, Switzerland) or anti-mouse Gr1 monoclonal antibody (BD Pharmingen, Franklin Lakes, NJ, USA) and visualized using the DAKO AEC substrate system (Agilent Technologies, Santa Clara, CA, USA) and hematoxylin (Sigma Chemical Co., St. Louis, MO, USA). Two blinded observers counted ER-HR3-positive cells (macrophages) and Gr-1-positive cells (neutrophils) on each slide in five random fields at a magnification of ×200.

### 4.6. Quantitative Real-Time PCR

Total RNA extraction from colon tissues was performed using a Qiagen mini kit (Qiagen, Valencia, CA, USA) and silica-membrane RNeasy spin columns, following the supplier’s instructions. Real-time quantitative PCR (qRT-PCR) was conducted using the iTaq universal SYBR Green I supermix (Bio-rad, Hercules, CA, USA) and primers for glyceraldehyde 3-phosphate dehydrogenase (GAPDH) as an internal control. The PCR primer sequences used were as follows: VEGF-C, sense: 5′- AGA CGG ACA CAC ATG GAG GT -3′ and antisense: 5′- AAA GAC TCA ATG CATGCC AC -3′; VEGF-D, sense: 5′- TTG AGC GAT CAT CCC GGT C -3′ and antisense: 5′- GCG TGA GTC CAT AGG GCA A -3′; VEGFR-3, sense: 5′- CTG GCA AAT GGT TAC TCC ATG A -3′ and antisense 5′- ACA ACC CGT GTG TCT TCA CTG -3′; IL-1β, sense: 5′- TGT AAT GAA AGA CGG CAC ACC -3′ and antisense: 5′- TCT TTG GGT ATT GCT TGG -3′; IL-6, sense: 5′- TGG AGT ACC ATA GCT ACC TGG A -3′ and antisense: 5′- TGA CTC CAG CTT ATC TGT TAG GAG -3′; IFN-γ, sense: 5′- ACT GGC AAA AGG ATG GTG A -3′ and antisense: 5′- GCT GTT GCT GAA GGT AG -3′; TNF-α, sense: 5′- ACC CTC ACA CTC AGA TCA TC -3′ and antisense: 5′- GAG TAG ACA AGG TAC AAC CC -3′; and GAPDH, sense: 5′-AAA TGG TGA AGC TCG CTC TG -3′ and GAPDH antisense: 5′-TGA AGG GGT CGT TGA TGG-3′.

### 4.7. Enzyme-Linked Immunosorbent Assay (ELISA)

Total protein extraction from frozen colon tissue was performed using radioimmunoprecipitation assay (RIPA) buffer (Elpis Biotechnology, Seoul, Korea) containing protease inhibitor cocktails (Sigma-Aldrich, St. Louis, MO, USA), and proetin levels were quantified. Levels of IL-1β, IL-6, IFN-γ, and TNF-α, in samples of equal protein concentration, were measured in triplicate using a Biolegend MAX ELISA Kit (San Diego, CA, USA).

### 4.8. Evans Blue Dye Assay

For lymphatic vessel flow measurement, the Evans blue dye assay was performed using a modified protocol [31,32]. Evans blue dye (1%) in normal saline (Sigma-Aldrich) was injected into the tail veins of the mice. After 30 min, the collected colon tissue was homogenized in formamide and then centrifuged at 16,000× *g* for 30 min at 4 °C. The absorbance of the recovered supernatant was recorded at 620 nm using a microplate reader (Molecular Devices, San Jose, CA, USA).

### 4.9. Statistical Analysis

All data are shown as the mean ± SD. Mean comparisons between two groups were assessed for significant differences using ANOVA, followed by individual comparisons with a Tukey’s post-hoc test; a *p*-value < 0.05 was considered significant.

## Supplementary Materials

Supplementary materials can be found at https://www.mdpi.com/1422-0067/21/21/8068/s1, Figure S1: Histopathological examination of colon tissue obtained 7 days after the commencement of DSS administration, stained with hematoxylin and eosin. Cont (no DSS; control), DSS (3% DSS), DSS + Art (3% DSS + artemisinin at 20 mg/kg body weight per day). Original magnification: 200×.

## Abbreviations

IBD	inflammatory bowel disease
DSS	dextran sulfate sodium
PECAM-1	platelet endothelial cell adhesion molecule-1
LYVE-1	lymphatic vessel endothelial hyaluronan receptor-1
DAI	disease activity index
LVD	lymphatic vessel density
VEGF	vascular endothelial growth factor
IL-1 β	interleukin-1β
IFN-γ	Interferon-γ
TNF-α	Tumor necrosis factor-α
