# Supplementary material for: The Effect of Artemisinin on Inflammation-Associated Lymphangiogenesis in Experimental Acute Colitis
Source: Int J Mol Sci. 2020 Oct 29;21(21):8068. doi: 10.3390/ijms21218068 (PMC7662347; doi:10.3390/ijms21218068)
Supplement: Supplementary file 1 [file ijms-21-08068-s001.pdf]

Supplementary Figure 1

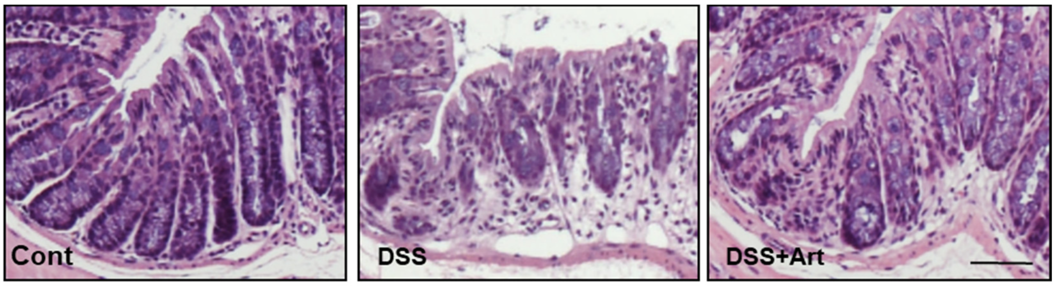

Supplementary Figure 1. Histopathological examination of colon tissue obtained 7 d after commencement of DSS administration and stained with hematoxylin and eosin. Cont (no DSS; control), DSS (3% DSS), DSS + Art (3% DSS + artemisinin at 20 mg /kg body weight per day). Original magnification: 200x.
